# Enhanced Tunability of Dual-Band Chiral Metasurface in the Mid-Infrared Range via Slotted Nanocircuit Design

**DOI:** 10.3390/nano14110979

**Published:** 2024-06-05

**Authors:** Shengyi Wang, Hanzhuo Kuang, Wenjie Li, Yanni Wang, Hao Luo, Chengjun Li, Hua Ge, Qiu Wang, Bowen Jia

**Affiliations:** 1 School of Information Engineering, Wuhan University of Technology, Wuhan 430070, China; shengyiwang@whut.edu.cn (S.W.); 301593@whut.edu.cn (H.K.); 288247@whut.edu.cn (W.L.); 275907@whut.edu.cn (H.L.); chengjunli@whut.edu.cn (C.L.); gehua@whut.edu.cn (H.G.); 2 Department of Respiratory and Critical Care Medicine, Renmin Hospital of Wuhan University, Wuhan 430060, China; wangyanni@whu.edu.cn

**Keywords:** mid-infrared, biochemical sensing, chiroptical metasurface, tunable dual-band

## Abstract

Multi-band circular dichroism (CD) response and tunability on the chiral metasurface are crucial for this device’s applications in sensing and detection. This work proposes a dual-band CD Au-CaF_2_-Au dimer elliptical metasurface absorber, where chiroptical sensing is realized by breaking the geometric symmetry between two ellipses. The proposed metasurface can achieve high CD values of 0.8 and −0.74 for the dual-band within the 3–5 μm region, and the CD values can be manipulated by independently adjusting the geometric parameters of the metasurface. Furthermore, a slotted nanocircuit is introduced onto the metasurface to enhance its tunability by manipulating the geometry parameter in the design process, and the related mechanism is explained using an equivalent circuit model. The simulation of the sensing model revealed that the slotted nanocircuit enhances the sensor’s tunability and significantly improves its bandwidth and sensitivity, achieving peak enhancements at approximately 753 nm and 1311 nm/RIU, respectively. Due to the strong dual-band positive (and negative) responses of the CD values, flexible wavelength tunability, and nonlinear sensitivity enhancement, this design provides a new approach for the development and application of mid-infrared chiroptical devices.

## 1. Introduction

The chirality of natural substances in the natural world has significant applications across various domains, such as pharmaceutical development [1,2], DNA structure sensing [3], and optical communication [4,5]. When illuminated by left circularly polarized (LCP) light and right circularly polarized (RCP) light, chiral matter produces varying absorption spectra leading to discernible CD responses [6], which has been a crucial characteristic of the analysis of matter in recent decades [7]. The chirality behavior of natural materials is constrained by the intrinsic chiral dielectric constant properties, which highly limited the chiroptical CD response [8]. Optical metasurfaces, serving as artificially tunable electromagnetic materials, have found extensive applications in chiral optics and electromagnetics [9,10,11]. A significant chiroptical CD response is obtained by leveraging the asymmetric structure of optical metasurfaces, as exemplified by letter-shaped patterns [12], split-ring resonators [13,14], and double-bar metasurfaces [15]. The high response of chiroptical properties promotes the utility of asymmetric metasurfaces in many scientific applications, such as in biosensing, infrared photodetection, and perfect absorbers [16,17,18,19,20]. Research has already been conducted on dual-band chiroptical metasurfaces. Tang et al. developed an Au-Al_2_O_3_-Au chiroptical metasurface for the selective absorption of CD in the 4–6 μm region [21]. Ouyang et al. proposed a broadband absorber with a chiroptical effect in the near-infrared region [22]. The ellipse metasurface is often used in the chiroptical sensing systems, and Zeng et al. presented a metasurface for CD spectra with split-ellipse structures in the 5–7 μm region [23]. Ali et al. presented elliptical nanoholes to achieve dual-band CD responses [24]. Carvalho et al. combined the magnetic circular dichroism, nanocavities and magneto-optical hyperbolic metamaterials to pave a new way to achieve the CD response on the metasurface [25]. It should be noted that most current research has not focused on dual-band chiroptical mid-infrared (MIR) sensing applications in the 3–5 μm region for CD, which includes the optical signals of hydroxyl (−OH) and alkyl (C−H) at 2.73–3.10 μm and 3.3–3.6 μm, respectively [26,27]. The dual-band characteristic is a pressing demand for enhancing the performance metrics of CD response devices.

Tunability is another crucial performance factor for sensing systems, as it can significantly improve their accuracy and practicality. Previous studies have primarily focused on scaling up the overall structure of metasurfaces [21] and using phase-transition materials [28] to achieve peak wavelength shifts. Though these two approaches can effectively tune the operating range, the process of changing the whole structure remains complex [12,21,23], and the intensity of resonance peaks generated by phase-change materials significantly decreases, which highly reduces their applicability for sensing systems [28,29]. A method involving structural alterations to form nanocircuits on nanoantennas has been proposed to effectively achieve tunability while maintaining the intensity of the shifted resonance peaks [30,31]. This method only requires simple structural modifications of the nanoantennas to achieve effective tunability on plasmonic and all-dielectric metasurfaces while preserving the original resonance peaks’ intensity [30,31,32,33,34,35]. This inspired us to design a slotted nanocircuit chiroptical metasurface to enhance CD sensing systems’ tunability by manipulating the geometric parameter in a unit cell in the design process.

In this paper, we propose an MIR chiroptical metasurface for CD sensing, which can generate a dual-band highly chiral selective response in the 3–5 μm region based on an Au-CaF_2_-Au metal–insulator–metal (MIM) structure. The dual-band selectivity results from the interaction of LCP and RCP light with the absorber originating from the symmetry breaking into two asymmetric dimer elliptical metal resonators integrated within the top layer of the Au metasurface. The independent control of the CD values obtained at various wavelengths is achieved via parameter adjustments within the metasurface. We also demonstrate a slotted nanocircuit method explained using a nanoantenna physical equivalent circuit model to improve the wavelength tunability instead of the mainstream “scale-up” technique or use of phase-transition materials. Additionally, the slotted nanocircuit was investigated on a sensing model and effectively achieved nonlinear enhancements in sensitivity and bandwidth, reaching maximums of 1311 nm/RIU and 753 nm, respectively. Due to the tunable strong CD response to incident MIR light exhibited by our proposed dual-band chiroptical metasurface, this device will experience extensive utility in MIR applications, including molecular biosensing, chiroptical photodetection, and free-space optical communication.

## 2. Structure and Design Methods

**Figure 1:**
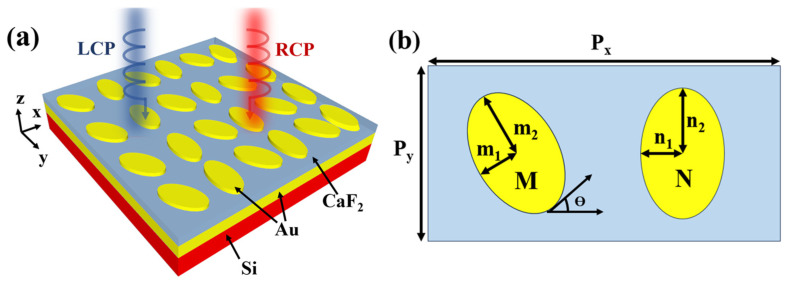
(**a**) Schematic of the Au-CaF_2_-Au chiroptical metasurface absorber. (**b**) The top view of the asymmetric elliptical metasurface.

Figure 1a shows the schematic of the Au-CaF_2_-Au dual-band chiral metasurface absorber, comprising a 1000 nm thick silicon substrate, a 400 nm thick Au bottom mirror, a 280 nm thick CaF_2_ spacer, and a 60 nm thick layer of metasurfaces composed of dimer elliptical Au resonators. Figure 1b shows the top view of the asymmetric dimer elliptical metasurface and its parameters. The major and minor radii of the slanted ellipse, rotated by an angle of ϴ (the numerical value of ϴ is selected in the simulation; see more details Supplement Document S1), are m_1_ and m_2_, respectively. The major and minor radii of the vertical ellipse are n_1_ and n_2_. This design disrupts the parity of the metasurface, leading to pronounced chiroptical responses under MIR light illumination. The resonances on both sides of the ellipses produce two resonance peaks in the absorption spectra, contributing to the dual-band chiroptical responses. The symmetry breaking in the dimer elliptical metasurface induces different E-field resonance modes from the incident LCP and RCP MIR light, resulting in varying resonance intensities for different CP lights. We detail the origin of the dual-band responses in the four structures we designed in the next paragraph. Because the MIR CP light cannot cross the Au mirror positioned at the bottom of the absorber, we calculate the normalized absorption of the chiral light using the equation A = 1 − R, where R represents the normalized reflectance of the incident MIR light.

Four types of Au-CaF_2_-Au dual-band chiral metasurface absorbers are proposed for different resonance mode states, which cause the chiroptical absorption of LCP and RCP light. We obtained four different CDs with these four structures. The geometric parameters of the four structures are shown in Table 1. We used the Lumerical finite-difference time-domain (FDTD) method to simulate the designed metasurface absorber’s absorption peaks at two wavelengths. Periodic boundary conditions are utilized in the x- and y-directions while a perfectly matched layer is implemented as a boundary condition in the z-direction to ensure the effective absorption of outgoing waves. The permittivity values of CaF_2_ and Au were provided by Malitson et al. [36] and Babar et al. [37], respectively.

## 3. Results and Discussion

**Figure 2:**
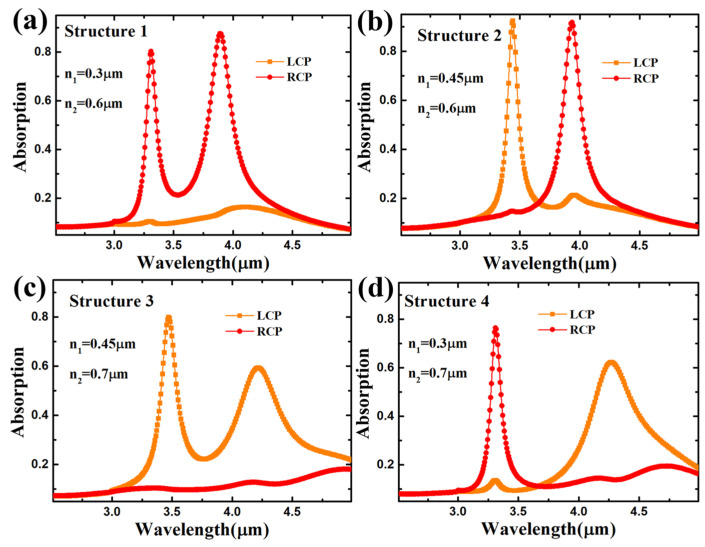
(**a**–**d**) Chiroptical absorption spectra for the Au-CaF_2_-Au chiroptical metasurface absorber with different geometric parameters shown in Table 1.

Figure 2a–d show the absorption spectra of the four metasurface absorbers separated by different geometric parameters. The spectra reveal four distinct absorption peak states, which were discernible by modulating the LCP and RCP light across the 3–5 μm range. The chiroptical resonant structure 1 is illustrated in Figure 2a, demonstrating a pronounced feature where the LCP light surpasses the RCP light’s intensity at both resonant peaks. Both the major and minor axes of structure M exceed those of structure N within structure 1. As per the definition of CD, the value of CD at the two resonant wavelengths is negative. This observation bolsters the credibility of the theoretical proposition that dipole moments induce alterations in various resonant modes in CD measurements. Figure 2b shows that distinct absorption peak sources emerge when diverse chiral light illuminates the metasurface by extending the n_1_ in structure N to 0.45 μm while maintaining the unchanged configuration of structure M and n_2_. The LCP and RCP light demonstrate resonances at 3.47 μm and 3.91 μm, respectively, yielding positive and negative CD values at these respective wavelengths. As shown in Figure 2c, we increased the n_1_ and n_2_ to 0.45 μm and 0.7 μm, respectively. The absorption of the two resonance peaks emitted by the LCP light is stronger than that of the RCP light. The CD spectra are positive at all wavelengths. As shown in Figure 2d, we changed the parameter of n_1_ back to 0.3 μm, and n_2_ remained unchanged. The metasurface shows a chiral response in structure 2, but the resonance strength of the RCP light at the shorter wavelength surpasses that of the LCP light at the longer wavelength. Thus, manipulating the geometric parameters of the comprehensive elliptical metasurface enables the realization of a dual-band, dual-absorption peak MIR chiral light absorber. The four structures exhibit unique responses to LCP and RCP light and substantial sensitivity to their respective characteristics. This underscores the potential utility of circular dichroism for differentiating the absorption spectra of distinct circularly polarized lights.

**Figure 3:**
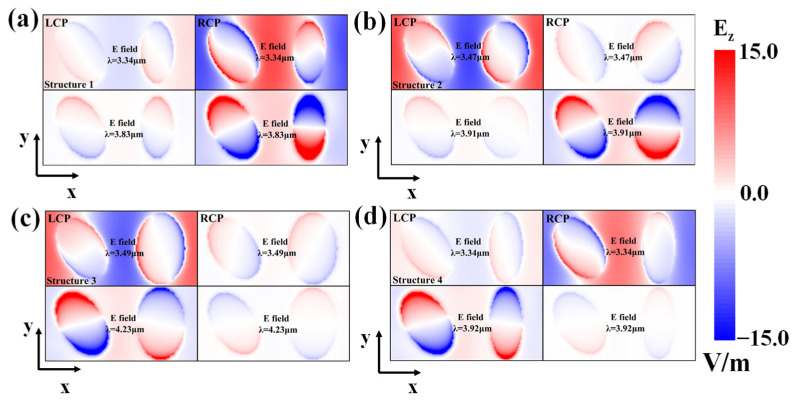
E_z_-field distribution generated by MIR LCP and RCP at both resonance peaks in (**a**) structure 1 (m_1_ > n_1_, m_2_ > n_2_), (**b**) structure 2 (m_1_ < n_1_, m_2_ > n_2_), (**c**) structure 3 (m_1_ < n_1_, m_2_ < n_2_), and (**d**) structure 4 (m_1_ < n_1_, m_2_ > n_2_).

Figure 3 displays the top view of the E_z_-field distributions at the interface of Au-CaF_2_ for both resonance peaks’ wavelengths of the chiroptical metasurface in the X–Y section. Figure 3a represents the resonance modes in the ellipses of structure 1 (m_1_ > n_1_; m_2_ > n_2_), in which LCP and RCP MIR generate peaks at 3.34 μm and 3.83 μm, respectively. The resonance modes differ between LCP and RCP light due to the geometric symmetry-breaking in the ellipses. The RCP light’s field intensity is significantly higher than the LCP light’s; hence, the RCP light’s resonance strength is significantly stronger than the LCP’s resonance strength. This means that RCP light causes stronger absorption peaks than LCP light at both resonance wavelengths. As shown in Figure 3b for structure 2 (m_1_ < n_1_; m_2_ > n_2_), peaks are generated by LCP and RCP MIR at 3.34 μm and 3.83 μm, respectively. The n_1_ exceeds the m_1_. The E_z_-field distributions show asymmetry in M and N that leads to different resonance modes for the LCP and RCP light, respectively. The RCP light in structure 2 generates a stronger field intensity at 3.91 μm than the LCP light due to the change in the symmetry-breaking by altering n_1_. While the field intensity generated by LCP light at 3.47 μm is stronger than that generated by RCP light, the field intensity could be equivalent to the resonance strength, causing two different CD responses at 3.47 μm and 3.91 μm in the MIR spectrum, respectively, as shown in Figure 2b. As shown in Figure 3c, peaks are generated in structure 3 by LCP and RCP MIR at 3.49 μm and 4.23 μm (m_1_ < n_1_; m_2_ < n_2_), respectively. The major and minor radii n_1_ and n_2_ are larger than m_1_ and m_2_. The coupling of the incident CP light and the resonance modes is similar to that in the first structure; conversely, the LCP light’s resonance strength is much higher than the RCP light’s, causing the LCP light’s absorption at the two resonance peaks to be larger than the RCP’s. Compared with the field distributions in Figure 3a,b, the resonance of the metasurface is weaker, which causes a weaker absorption peak for the LCP light in structure 3 than it does for the RCP light in structure 1. As shown in Figure 3d, peaks are generated by the LCP and RCP light at 3.34 μm and 3.92 μm in structure 4 (m_1_ < n_1_; m_2_ > n_2_), respectively. The E_z_-field distribution’s asymmetry characteristic is similar to that in structure 2. As shown in Figure 2d, the RCP light shows a stronger field intensity and resonance strength at 3.34 μm than the LCP light, which causes the resonance peak in the CD spectrum. Meanwhile, the RCP light exhibits a higher resonance strength at 3.92 μm, which causes another resonance peak in the CD spectrum.

Based on the E_z_-field distribution of all structures, it can be determined that the geometric symmetry-breaking on the dimer elliptical metasurface generates the different CD responses. When changing one of the major or minor radii in the ellipse M or N, the CD value transforms the different absorption peaks generated by the different CP lights in the spectrum. The next paragraph will discuss the CD value’s relation to the geometric parameter.

The value of CD is calculated using Equation (1) in this work, where *A_LCP_* and *A_RCP_* represent the normalized absorption of the LCP light and the RCP light, respectively [21,22,23].

(1) $$CD=A_{LCP}-A_{RCP}$$

**Figure 4:**
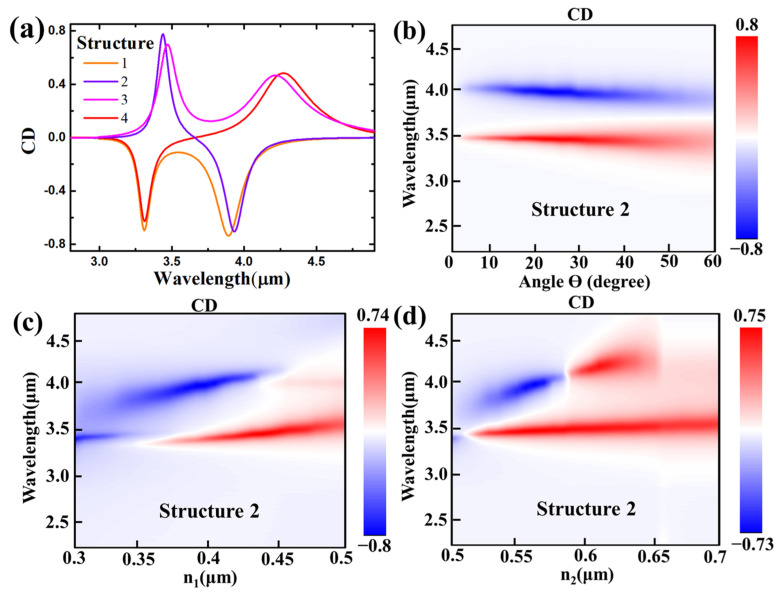
(**a**) CD spectra for four metasurface structures. The CD spectra mapping for Structure 2 as a function of the value of the geometric parameter (**b**) ϴ, (**c**) n_1_ and (**d**) n_2_.

Circular dichroism exhibits a positive value when the metasurface’s absorption for LCP light surpasses that for RCP light; conversely, it takes a negative value when the LCP light’s absorption rate is inferior to the RCP’s. The CD spectrum based on Figure 2 is presented in Figure 4a. In our work, the CD value at the resonance peak is approximately 0.8, and the dual-band characteristic is shown in Figure 4a. We can obtain structures that exhibit discernible positive and negative CD values by independently manipulating the geometric parameters of M and N within the metasurface. Here, we use structure 2, which has two opposite strong CD values in the spectrum, as an example. As shown in Figure 4b, the CD spectra for the rotation angle of ellipse M consistently display two CD values with opposing signs at approximately 3.5 μm and 4 μm, irrespective of the adjustment in rotation angle. The resonant wavelengths remain nearly invariant despite alterations in the rotation angle. The CD values at the resonance peaks for the LCP and RCP light converge at around 0.8. The geometric parameter n_1_ increases from 0.3 μm to 0.5 μm in Figure 4c. Compared with previous works [21,22,23], our metasurface consistently has no transition point as the n_1_ varies. The designed dimer elliptical metasurface can thus prevent a zero CD value point during the n_1_-varying process. The geometric parameter n_2_ increases from 0.5 μm to 0.8 μm in Figure 4d. Unlike the variation in n_1_, n_2_ shows a transition point when the length of n_2_ is equal to 0.59 μm. At this point, the structure absorbs the LCP and RCP incident light nearly equally, resulting in a zero CD value in the spectrum. The CD spectrum mapping for structure 2 as a function of the CD value via the geometric parameter provides strong evidence that the metasurface preserves its dual-band attributes without undergoing alterations across variations in the structural parameters. Furthermore, the CD values are consistent with their initial characteristics, consistently hovering around 0.8, which is a stable and attractive characteristic for CD sensing applications.

**Figure 5:**
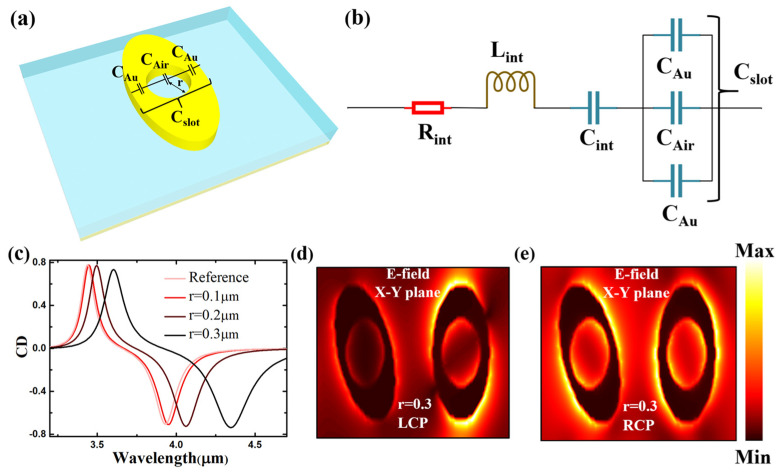
(**a**) Schematic of the CD slotted metasurface structures. (**b**) Equivalent circuit model. (**c**) The CD value of the metasurface by changing the radius of the slot in structure 2. The E-field distribution in the X–Y plane when the radius of the slot is r = 0.3 μm for the incident (**d**) LCP and (**e**) RCP.

A method to enhance the wavelength’s tunability is proposed, where slotted nanocircuits are introduced into the resonators. This method can further explain the origination of the wavelength shifts in the two resonance peaks in the CD spectrum. This method originates from the E-field’s continuity at high-index-contrast interfaces and has been widely used to expose and enhance electromagnetic fields, which are distributed in high-index materials [38,39,40]. In our work, the slots in the resonator form a cylinder, where r is the radius, as shown in Figure 5a. The nanostructure in the slotted resonator can be considered an equivalent circuit model, as shown in Figure 5b. Our model is based on the optical nanoantenna theory, which treats the metasurface as a dipolar antenna under light illumination [30,31,32,33,34,35]. For the approximation, we assume that the resonator only exhibits radiative loss and is positioned in a homogeneous background (i.e., air, in our theory). When only considering the radiative loss, the single elliptical resonator on the metasurface can be described using an RLC circuit, with the intrinsic impedance of the whole CD metasurface system expressed as Equation (2) [41]:

(2) $$Z_{int}=R_{int}-{jX}_{int}=R_{int}-{j\omega L}_{int}-\frac{1}{{j\omega C}_{int}}$$

where j denotes the imaginary unit, and *R_int_* is the intrinsic resistance, which is denoted by the radiative loss. As illustrated in Figure 5a, the slotted resonator can be considered a circuit containing multiple capacitors in series when it is excited by the MIR CP light [31,32]. Both the original “no-slot” and slotted resonators are analyzed using the equivalent circuit model to emphasize the role of the slots. The original “no-slot” resonator is considered as a nanoantenna with gaps loaded with Au, as shown in Figure 1b. The load capacitance of the nanoantenna can be expressed as shown in Equation (3) [30,31,34]:

(3) $$C_{original}={8\varepsilon }_{Au}\varepsilon_{0}hm_{1}(or\;n_{1})/\pi r$$

where *ε_Au_* (numerically, −515~−1169 in the 3~5 μm region), *ε*_0_ denotes the Au’s permittivity and the background (filled with air in this work), and h and r are the height (60 nm) and the radius of the slots in our design, as shown in Figure 5a. Each slot in the resonator can be considered a parallel connection between *C_Au_* and *C_Air_* in the equivalent circuit model, as shown in Figure 5a,b. The load capacitance of the slot in the equivalent circuit can be obtained using Equation (4):

(4) $$C_{slot}=(4\varepsilon_{0}\varepsilon_{Au}h\left(2m_{1}\left(or\;n_{1}\right)-\frac{\pi }{4}r\right)+\frac{\pi }{4}r)/\pi r$$

when *r* = 0, and the two capacitances contribute equally. The resonance peak of the metasurface is equal to the open-circuit resonant frequency *ω*_0_, which can be calculated using Equation (5):

(5) $$\omega_{0}=1/X_{int}C_{slot}$$

By combining Equations (4) and (5), the open-circuit resonant frequency *ω*_0_ can be expressed using Equation (6):

(6) $$\omega_{0}=1/{(X}_{int}\left(\frac{8m_{1}(or\;n_{1})}{\pi r}-1\right)\varepsilon_{0}\varepsilon_{Au}h+\frac{1}{4})$$

where *r* is considered the argument of Equation (6). In our slotted resonator, *r* is always numerically smaller than m_1_, so $\frac{8m_{1}}{\pi r}-1$ is always a positive value. Because the *ε_Au_* is numerically −515~−1169 in the 3–5 μm region, the $X_{int}\left(\frac{8m_{1}}{\pi r}-1\right)\varepsilon_{0}\varepsilon_{Au}h+\frac{1}{4}$ exhibits an increasing trend when the *r* increases. This causes the resonance peaks to show a redshift trend in the CD’s wavelength spectra. n_1_ > m_1_ in our structure 2 model due to the difference in the m_1_ and n_1_ in the two resonators on our proposed metasurface. This causes the influence of the variation in r on $\frac{8n_{1}}{\pi r}$ to be larger than on $\frac{8m_{1}}{\pi r}$ and leads to the different variations between the resonant frequency ω_0_ in the two resonators. Additionally, this causes the difference between the redshift in the two resonance peaks in the CD spectra, as shown in Figure 5c.

The Q-factor in the CD spectra can also be explained via the equivalent circuit model. For the RLC circuit, the Q-factor can be calculated with Equation (7):

(7) $$Q=R_{int}^{-1}\sqrt{L_{int}/C_{whole}}\propto 1/C_{whole}$$

where the $C_{whole}$ is given by $C_{whole}={\left(\frac{1}{C_{int}}+\frac{1}{C_{slot}}\right)}^{-1},$ as shown in the circuit model in Figure 5b. The $C_{slot}$ increases due to the increase in the r, which is due to the $C_{whole}$ increase. This leads to the decrease in the Q-factor of the resonance peaks in the CD spectrum, as shown in Figure 5c.

The E-field distribution in the X–Y plane illuminated by the LCP and RCP light is shown in Figure 5d,e. The resonance in the slotted resonator is outside the resonator as well as inside the slot edges. The different E-field distributions between the slotted resonator illuminated by the LCP and RCP light cause the dual-band CD response in the spectra and maintain the CD value’s strength. This strategy realizes the metasurface’s tunability by tuning the two resonance modes, unlike the mainstream “scale-up” technique and using phase transition materials [21,28,29].

The proposed symmetry-breaking dimer elliptical metasurface is characterized by dual-band tunability, which holds potential for use in refractive sensing applications such as CP light filtering and absorption. The wavelength of the dual-band CD responses highly depends on the surrounding medium, which provides an efficient method for gas and liquid sensing applications. A dimer elliptical metasurface-based sensor was designed for the sensitivity simulation. The metasurface’s parameters were set as P_x_ = 3 μm, P_y_ = 1.5 μm, θ = 30°, m_1_ = 0.4 μm, m_2_ = 0.65 μm, n_1_ = 0.3 μm, and n_2_ = 0.6 μm. The sensing performance can be evaluated by the sensitivity, which indicates how much the peak’s wavelength shifts when the refractive index changes, that can be defined as Equation (8):

(8) S=∆λ/∆n

where Δλ represents the wavelength shift of the peak, and Δn represents the refractive index’s variation in the matter on the top of the metasurface.

**Figure 6:**
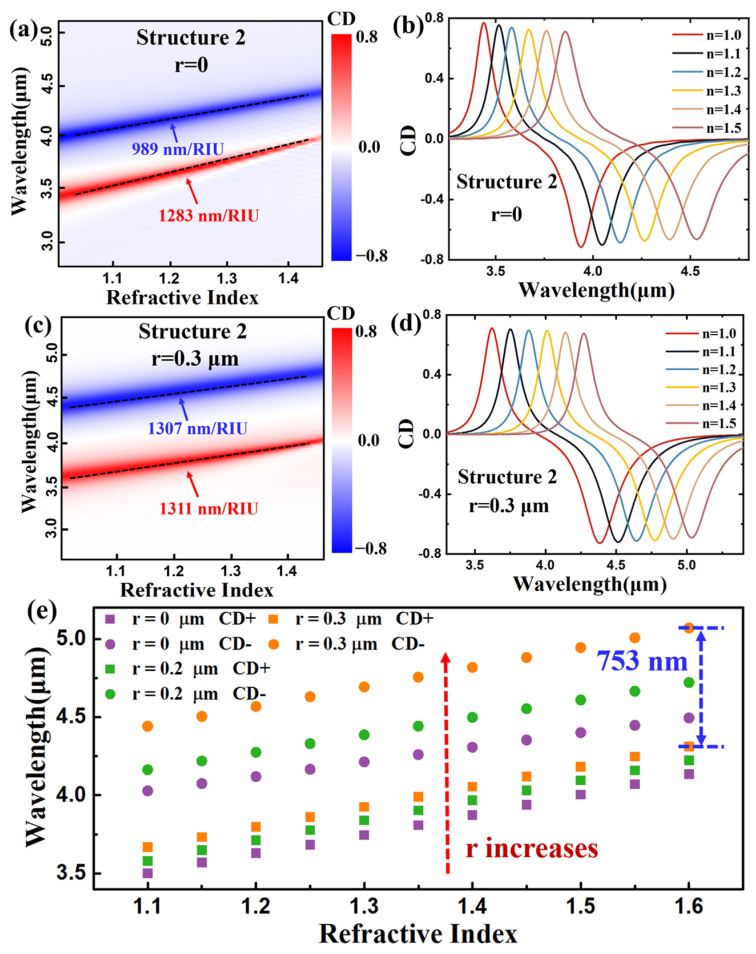
CD spectrum maps of structure 2 (**a**) without slotted nanocircuit, (**c**) with r = 0.3 μm slotted nanocircuit. CD spectrum via wavelength under different refractive index of the surrounding medium of structure 2 (**b**) without slotted nanocircuit, (**d**) with r = 0.3 μm slotted nanocircuit. (**e**) The location of CD resonance peaks via different refractive index of the surrounding medium of slotted nanocircuit with different radius.

Figure 6a,c show CD spectrum maps showing a refractive index from 1 to 1.5 for structure 2 without and with a slotted nanocircuit (r = 0.3 μm), respectively. Both peaks approach a linear relationship as the refractive index increases, undergoing peak shifts. The negative CD peak with sensitivity S = 989 nm/RIU is significantly smaller than the positive CD peak with S = 1283 nm/RIU in structure 2 without a nanocircuit; meanwhile, the negative CD peak with sensitivity S = 1307 nm/RIU is nearly the same as the positive CD peak with S = 1311 nm/RIU with a slotted nanocircuit (r = 0.3 μm). The CD spectrum with wavelengths in different surrounding mediums was also studied, as shown in Figure 6b,d. Both CD peaks in the spectrum can maintain consistently high CD values as the refractive index changes from 1 to 1.5. Additionally, the peak shifts with a slotted nanocircuit (r = 0.3 μm) are relatively larger than those without one. The relation of different slotted radii with the peak locations in the CD spectrum was also investigated, as shown in Figure 6e. Both positive and negative CD peak values undergo a redshift as the radius increases. The redshift of the positive CD peak value is relatively stable, while that of the negative CD peak value continues to increase. This result is consistent with the conclusions observed in Figure 6a,c. Furthermore, the sensitivity of the negative CD peak value gradually improves as the radius increases, showing a nonlinear growth trend. The distance between the two peaks also shows an increasing trend, thereby achieving a higher bandwidth, which exceeds 753 nm at the maximum, as shown in Figure 6e. This investigation proves that our slotted nanocircuit method effectively achieves a wide range of peak tunability for CD resonance peaks and enhances the sensitivity and bandwidth of the dimer elliptical metasurface.

Finally, we compare the performance of our dual-band dimer elliptical metasurface with those of previous studies, as listed in Table 2. Many prior studies have utilized the MIM structure, exhibiting single-band CD spectra due to the influence of geometric symmetry-breaking metasurface shapes, which leads to distinct LCP and RCP light absorption responses at different wavelengths. Table 2 also displays the CD responses of our chiroptical metasurface, which exceed those of recently reported metasurfaces operating in the MIR region and exhibit a balance between the maximum and minimum CD values. In short, the metasurface in our work demonstrates a strong dual-band CD response in the 3–5 µm MIR region, a spectral range that has not been extensively explored. This metasurface, enhanced by its flexible tunability via a slotted nanocircuit, holds potential for impactful applications in MIR CD sensing.

## 4. Conclusions

In this work, we proposed a dual-band MIR chiroptical metasurface with a high CD value. The symmetry-breaking design of the two ellipse Au resonators on the top Au layer enables the dual-band CD’s selectivity and tunability in the chiroptical metasurface absorbers when illuminated by LCP and RCP MIR light. The CD value in this study exceeded 0.81. The CD spectra were manipulated by adjusting the interactions between the two elliptical Au resonators via straightforward modifications of their geometric attributes, specifically the major and minor radii of the vertically oriented ellipse (referred to as structure N in this work). The investigation of the CD spectra mapping for structure 2 indicated the dependence of the CD value on metasurfaces’ geometric parameters. Moreover, we introduced a slotted nanocircuit to enhance the wavelength tunability in the 3~5 μm MIR CD spectrum, which was explained with a physical equivalent circuit model. Our investigation of the sensing model revealed that the slotted nanocircuit maintained the strength of resonance peaks more effectively than the traditional “scale-up” technique and use of phase-transition materials and provided sensitivity and bandwidth enhancements, where the sensitivity exceeded 1311 nm/RIU and the bandwidth reached 753 nm. The high CD value and dual-band tunability make our design an important candidate for many applications in the MIR chiroptical field, such as biomedical diagnostics, environmental monitoring, and food analysis.

## Figures and Tables

**Table 1:** Basic parameters of the dual-band chiral metasurface.

Structure	P_x_ (μm)	P_y_ (μm)	ϴ (°)	m_1_ (μm)	m_2_ (μm)	n_1_ (μm)	n_2_ (μm)
1	3	1.5	30	0.4	0.65	0.3	0.6
2	3	1.5	30	A0.4	0.65	0.45	0.6
3	2.9	1.5	31	0.4	0.65	0.45	0.7
4	2.9	1.5	31	0.4	0.65	0.3	0.7

**Table 2:** Comparison of chiroptical CD metasurface in other works.

Reference	Structure	CD Spectra	Incident Angle (°)	Region (μm)	Maximum CD	Minimum CD
[21]	MIM	Dual-band	90	4–6	0.6	−0.55
[42]	Enantiomer	Single-band	90	4–7	0.85	−0.41
[23]	MIM	Single-band	90	5–5.9	0.67	−0.53
[28]	MIM	Single-band	90	2–3	0.51	−0.44
[43]	MIM	Single-band	90	0.9–1	0.65	−0.45
[44]	Ag-SiO_2_	Multi-band	60	0.75–1	0.98	−0.95
[22]	MIM	Broadband	90	1.2–2	0.6	−0.6
[29]	BIC	Dual-band	90	2–2.5	0.98	−0.7
[45]	BIC	Single-band	90	0.9–1	0.99	−0.55
This work	MIM	Dual-band	90	3–5	0.8	−0.74

## Data Availability

The data presented in the study are available on request from the corresponding author. The data are not publicly available due to technical, resource, and time constraints.

## Supplementary Materials

The following supporting information can be downloaded at: https://www.mdpi.com/article/10.3390/nano14110979/s1, Figure S1. Absorption spectra with different tuning angles in (a) structure 2 and (b) structure 4. Reference [46] are cited in the supplementary materials.
