# Supplementary material for: Enhanced Tunability of Dual-Band Chiral Metasurface in the Mid-Infrared Range via Slotted Nanocircuit Design
Source: Nanomaterials (Basel). 2024 Jun 5;14(11):979. doi: 10.3390/nano14110979 (PMC11173487; doi:10.3390/nano14110979)
Supplement: Supplementary file 1 [file nanomaterials-14-00979-s001.zip › nanomaterials-3016400-supplementary.pdf]

Supplementary Information

Enhanced Tunability of Dual-Band Chiral Metasurface in the Mid-Infrared Range via Slotted Nanocircuit Design

Shengyi Wang [1,†], Hanzhuo Kuang [1,†], Wenjie Li [1], Yanni Wang [2], Hao Luo [1], Chengjun Li [1], Hua Ge [1], Qiu Wang [1,*] and Bowen Jia [1,*]

For extrinsic chiroptical metasurface systems, chirality arises from the breaking of spatial inversion symmetry, which requires rotational symmetry breaking. In our study, this is represented by the rotational angle theta of the elliptical resonator. Unlike the symmetry breaking in Bound States in the Continuum metasurfaces [S1], in typical chiral systems, the magnitude of chirality does not increase with decreased symmetry breaking. Instead, chirality peaks at a moderate level of symmetry breaking, specifically at angles of 30 and 31 degrees in our findings. This is demonstrated by the absorption spectra at various tuning angles, as shown in Fig. S1.

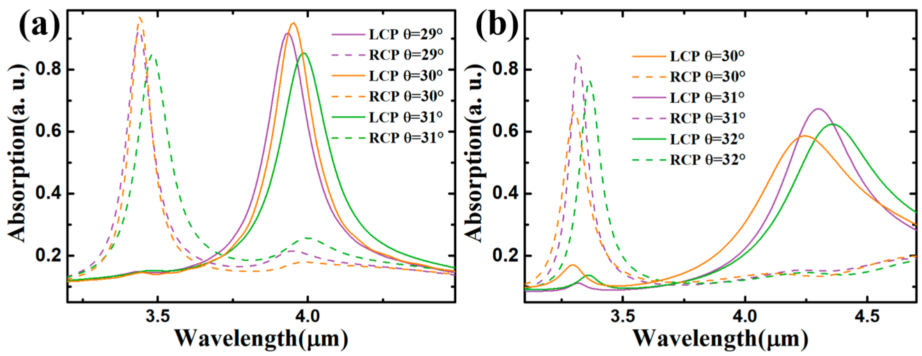

Fig. S1. Absorption spectra with different tuning angles in (a) structure 2 and (b) structure 4.

In structure 2, as theta increases, the amplitude of the resonance peak initially rises and then falls, with a clear maximum in occurring around theta equal to 30°. Likewise, in structure 4, the amplitude of the resonance peak also increases and then decreases with a clear circular dichroism maximum around theta equal to 31°. These observations led us to select theta values of 30° and 31° for the metasurface design.

References:

1. Y. Zhang, D. Chen, W. Ma, S. You, J. Zhang, M. Fan, C. Zhou, Active optical modulation of quasi-BICs in Si–VO_2 hybrid metasurfaces, Opt. Lett. 47 (21) (2022), 5517-5520.
